# Circulating cell-free DNA in health and disease — the relationship to health behaviours, ageing phenotypes and metabolomics

**DOI:** 10.1007/s11357-022-00590-8

**Published:** 2022-07-21

**Authors:** Laura Kananen, Mikko Hurme, Alexander Bürkle, Maria Moreno-Villanueva, Jürgen Bernhardt, Florence Debacq-Chainiaux, Beatrix Grubeck-Loebenstein, Marco Malavolta, Andrea Basso, Francesco Piacenza, Sebastiano Collino, Efstathios S. Gonos, Ewa Sikora, Daniela Gradinaru, Eugene H. J. M. Jansen, Martijn E. T. Dollé, Michel Salmon, Wolfgang Stuetz, Daniela Weber, Tilman Grune, Nicolle Breusing, Andreas Simm, Miriam Capri, Claudio Franceschi, Eline Slagboom, Duncan Talbot, Claude Libert, Jani Raitanen, Seppo Koskinen, Tommi Härkänen, Sari Stenholm, Mika Ala-Korpela, Terho Lehtimäki, Olli T. Raitakari, Olavi Ukkola, Mika Kähönen, Marja Jylhä, Juulia Jylhävä

**Affiliations:** 1 Department of Medical Epidemiology and Biostatistics, Karolinska Institutet, Stockholm, Sweden; 2 Faculty of Social Sciences (Health Sciences), and Gerontology Research Center, Tampere University, Tampere, Finland; 3 Faculty of Medicine and Health Technology, and Gerontology Research Center, Tampere University, Tampere, Finland; 4 Molecular Toxicology Group, University of Konstanz, Konstanz, Germany; 5 BioTeSys GmbH, 73728 Esslingen, Germany; 6 URBC-Narilis, University of Namur, Rue de Bruxelles, 61, B-5000 Namur, Belgium; 7 Research Institute for Biomedical Aging Research, University of Innsbruck, Rennweg, 10, 6020 Innsbruck, Austria; 8 Advanced Technology Center for Aging Research, Scientific Technological Area, IRCCS INRCA, Ancona, Italy; 9 Nestlé Research, Nestlé Institute of Health Sciences, EPFL Innovation Park, 1015 Lausanne, Switzerland; 10 Institute of Biology, Medicinal Chemistry and Biotechnology, National Hellenic Research Foundation, Athens, Greece; 11 Laboratory of the Molecular Bases of Ageing, Nencki Institute of Experimental Biology, Polish Academy of Sciences, 3 Pasteur street, 02-093 Warsaw, Poland; 12 Department of Biochemistry, Faculty of Pharmacy, “Carol Davila” University of Medicine and Pharmacy, 020956 Bucharest, Romania; 13 National Institute for Public Health and the Environment (RIVM), Centre for Health Protection, P.O. Box 1, 3720 BA Bilthoven, The Netherlands; 14 Straticell, Science Park Crealys, Rue Jean Sonet 10, 5032 Les Isnes, Belgium; 15 Institute of Nutritional Sciences (140), University of Hohenheim, 70593 Stuttgart, Germany; 16 Department of Molecular Toxicology, German Institute of Human Nutrition Potsdam-Rehbruecke (DIfE), Nuthetal, Germany; 17 Department of Physiological Chemistry, Faculty of Chemistry, University of Vienna, 1090 Vienna, Austria; 18 Institute of Nutritional Medicine (180), University of Hohenheim, 70593 Stuttgart, Germany; 19 Department of Cardiothoracic Surgery, University Hospital Halle, Ernst-Grube Str. 40, 06120 Halle (Saale), Germany; 20 DIMES- Department of Experimental, Diagnostic and Specialty Medicine, Interdepartmental Center “Alma Mater Research Institute On Global Challenges and Climate Change (Alma Climate)”, Alma Mater Studiorum, University of Bologna, 40126 Bologna, Italy; 21 Section of Molecular Epidemiology, Leiden University Medical Centre, Leiden, The Netherlands; 22 Unilever Science and Technology, Beauty and Personal Care, Sharnbrook, UK; 23 Center for Inflammation Research, VIB, Ghent, Belgium; 24 Department of Biomedical Molecular Biology, Ghent University, Ghent, Belgium; 25 National Institute for Health and Welfare, Helsinki, Finland; 26 Department of Public Health, University of Turku and Turku University Hospital, Turku, Finland; 27 Centre for Population Health Research, University of Turku and Turku University Hospital, Turku, Finland; 28 Computational Medicine, Faculty of Medicine, University of Oulu and Biocenter Oulu, Oulu, Finland; 29 Center for Life Course Health Research, University of Oulu, Oulu, Finland; 30 NMR Metabolomics Laboratory, School of Pharmacy, University of Eastern Finland, Kuopio, Finland; 31 Department of Clinical Chemistry, Faculty of Medicine and Health Technology, Tampere University, Tampere, Finland; 32 Finnish Cardiovascular Research Center, Faculty of Medicine and Health Technology, Tampere University, Tampere, Finland; 33 Department of Clinical Chemistry, Fimlab Laboratories, Tampere, Finland; 34 Research Centre of Applied and Preventive Cardiovascular Medicine, University of Turku, Turku, Finland; 35 Department of Clinical Physiology and Nuclear Medicine, Turku University Hospital, Turku, Finland; 36 Research Unit of Internal Medicine, Medical Research Center Oulu, Oulu University Hospital, University of Oulu, Oulu, Finland; 37 Department of Clinical Physiology, Tampere University Hospital, Tampere, Finland

**Keywords:** Cell-free DNA, Biomarker of ageing, Metabolomics, Health behaviours, Morbidity, Frailty

## Abstract

Circulating cell-free DNA (cf-DNA) has emerged as a promising biomarker of ageing, tissue damage and cellular stress. However, less is known about health behaviours, ageing phenotypes and metabolic processes that lead to elevated cf-DNA levels. We sought to analyse the relationship of circulating cf-DNA level to age, sex, smoking, physical activity, vegetable consumption, ageing phenotypes (physical functioning, the number of diseases, frailty) and an extensive panel of biomarkers including blood and urine metabolites and inflammatory markers in three human cohorts (N = 5385; 17–82 years). The relationships were assessed using correlation statistics, and linear and penalised regressions (the Lasso), also stratified by sex.

cf-DNA levels were significantly higher in men than in women, and especially in middle-aged men and women who smoke, and in older more frail individuals. Correlation statistics of biomarker data showed that cf-DNA level was higher with elevated inflammation (C-reactive protein, interleukin-6), and higher levels of homocysteine, and proportion of red blood cells and lower levels of ascorbic acid. Inflammation (C-reactive protein, glycoprotein acetylation), amino acids (isoleucine, leucine, tyrosine), and ketogenesis (3-hydroxybutyrate) were included in the cf-DNA level-related biomarker profiles in at least two of the cohorts.

In conclusion, circulating cf-DNA level is different by sex, and related to health behaviour, health decline and metabolic processes common in health and disease. These results can inform future studies where epidemiological and biological pathways of cf-DNA are to be analysed in details, and for studies evaluating cf-DNA as a potential clinical marker.

**Supplementary Information:**

The online version contains supplementary material available at 10.1007/s11357-022-00590-8.

## Introduction

Circulating cell-free DNA (cf-DNA) has emerged as a valid mortality predictor [1, 2] and a biomarker that provides information on many health [3, 4] and age-related conditions [5]. It can be used to monitor the progression and severity of various diseases, such as sepsis [6–8], trauma [9], cardiovascular diseases (CVDs) [10–12], acute viral infections [13, 14] and cancer [15]. cf-DNA levels are also increased in association with ageing-associated physiological changes and frailty [16], low-grade chronic inflammation [16, 17], and unfavourable lipid profile and high blood pressure [17]. The cf-DNA level can also transiently increase as a short-term response to emotional stress [18], physical exercise [19], and psychophysiological stress [20].

The level of cf-DNA in circulation depends on the balance between its release from cells and the clearance rate. cf-DNA originates from apoptosis, cell lysis, necrotic cell death and pathogen-clearance-system termed NETosis, the latter being characteristic to neutrophils in which net-like structures of chromatin and proteases are released to bloodstream [21]. In healthy individuals, the vast majority of cf-DNA originates from blood cells, endothelial cells and hepatocytes, whereas in disease states, pathological tissues contribute to the pool of circulating cf-DNA [22, 23]. The potential mechanisms underlying cf-DNA clearance include active uptake by the reticuloendothelial system in the liver and spleen, passive filtration by the renal system, and direct degradation by nucleases [24]. In addition, findings from a genome-wide genetic association analysis in healthy individuals imply that UGT1A1-enzyme-associated processes might be involved in the regulation of serum cf-DNA level. Genetics, however, seems to influence the cf-DNA level only modestly [25].

Despite the growing body of evidence demonstrating the usefulness of cf-DNA in risk stratification and monitoring disease progression, understanding on health and ageing-related factors and metabolic processes associated with baseline cf-DNA levels is lagging behind. Therefore, in this explorative analysis, we aimed to (i) identify the health factors, health behaviours and ageing phenotypes (i.e. age, sex, smoking, vegetable consumption, physical activity, physical functioning, the number of diseases, and frailty) that are related to the circulating cf-DNA levels, and (ii) assess the blood and urine biomarkers that are independently related to the cf-DNA levels, thus providing a biomarker profile for the cf-DNA. The analysis was performed in three cohorts: the MARK-AGE study, the Young Finns study (YFS) and the Health 2000 survey, together comprising ~ 5800 individuals (aged 17–82 years) with hundreds of biomarkers and metabolites available. The results provide the scientific community a large catalogue of associations between the cf-DNA levels and health-related factors relevant for population ageing, facilitating further studies into cf-DNA. The biomarker profile for the cf-DNA sheds light into the biological underpinnings of the circulating cf-DNA level.

## Methods

### Analytical samples

In this study, three cohorts with cross-sectional data, the MARK-AGE, the YFS, and the Health 2000 Survey were used (Table 1). MARK-AGE is European Study for characterisation of biomarkers of human ageing [26]. In MARK-AGE, data were collected between 2008 and 2012 in Germany, Belgium, Austria, Greece, Poland, Italy, Finland, and the Netherlands, and 2261 individuals aged 17–82 years were used in this analysis. The YFS is an ongoing follow-up study in Finland that has been set up for characterisation of cardiovascular risk factors [27]. The data used in this analysis were collected in 2001 (N = 1928, aged 24–39 years). The Finnish Health 2000 Survey (N = 8028, aged 30–80 + years) was conducted nationwide in 2000–2001 [28], and a subsample (N = 1196, aged 46–76 years) of the Health 2000, to which this study is based on, was recruited in 2001–2003.

**Table 1:** Characteristics of the analytical samples

	MARK-AGE		The Young Finns study		The Health 2000 Survey	
n	2261		1928		1196	
cf-DNA, ug/ml, median (IQR)	0.70 (0.15)		1.05 (0.24)		0.841 (0.14)	
Age range (median, IQR)	17–82 (60, 17)		24–39 (33, 9)		46–76 (57, 12)	
Women, n (%)	1225 (54.2%)		1072 (55.6%)		683 (57.1%)	
Smoking, n (%)	365 (16.1%)		441 (22.9%)		220 (18.4%)	
Vegetable consumption, n (%)						
	Never	2 (0.1%)	Less than once/month	45 (2.3%)	Never	64 (5.3%)
	1–3 times/month	29 (1.3%)	1–2 times/month	135 (7.0%)	1–2 days/week	121 (10.1%)
	1–3 times/week	285 (12.6%)	Once/week	159 (8.2%)	3–5 days/week	215 (18.0%)
	4–6 times/week	450 (19.9%)	Twice/week	343 (17.8%)	6–7 days/week	796 (66.6%)
	Every day	1123 (49.7%)	Almost daily	696 (36.1%)		
	Several times/day	372 (16.4%)	Once or several times/day	550 (28.5%)		
Physical activity						
	na		MET, median (IQR)	11.8 (28.3)	Low	407 (34.0%)
					Modest	347 (29.0%)
					Good level	384 (32.1%)
					High level	58 (4.8%)
Physical performance, Health limits to run 0.5 km						
	No	982 (43.4%)	na		No	561 (46.9%)
	A little	904 (40.0%)			A little	224 (18.7%)
	A lot	375 (16.6%)			A lot	91 (7.6%)
					Completely	320 (26.8%)
Number of diseases, n (%)						
0	1084 (47.9)				489 (40.9)	
1	721 (31.9)				393 (32.9)	
2	299 (13.2)				201 (16.8)	
3 +	157 (6.9)				113 (9.4)	
Frailty index, median (IQR)	0.12 (0.13)		na		na	

Abbreviations: *IQR*, interquartile range, *MET*, metabolic equivalent of task, *na*, not available for analysis

### Questionnaire and in-person-interview data

Sex, age, health behaviour (smoking, vegetable consumption, physical activity), and ageing phenotypes (physical functioning, number of diseases and frailty) were obtained from questionnaire and in-person interview data that are described in Table 1, and in Online Resource 1.pdf, Table S1 and Table S2. Physical activity was available only in the YFS and Health 2000, whereas physical functioning and the number of diseases were available only in MARK-AGE and Health 2000. Frailty index was available only in MARK-AGE.

Briefly, the number of diseases, i.e., the sum of diseases present in an individual, was calculated based on ten chronic disease diagnoses. In specific, the calculation was performed using binary indicators of asthma/chronic obstructive pulmonary disease, arthritis (including osteoarthritis or rheumatism), osteoporosis, heart failure, angina pectoris, hypertension, diabetes, cancer/tumour (malignant), infarction, and stroke, (cerebral thrombosis/haemorrhage), each giving one point if the diagnosis was present. The frailty index was based on the Rockwood deficit accumulation model and calculated according to a standard procedure [29] as the sum of the 39 items (described in Table S2 in the Online Resource 1.pdf) divided by 39. The items represent health-related deficits, such as diseases, physical functioning, symptoms, self-rated health, and psychosocial well-being.

### Biochemical analyses

The biomarkers were measured in blood samples in the YFS [30–33] and the Health 2000 [28, 34–37], whereas MARK-AGE had both blood and urinary biomarkers available [26, 38–40]. In the MARK-AGE, participants were instructed to fast overnight before sampling, in the YFS, at least 4 h and in the Health 2000, 10–12 h except if the participant had diabetes and was using insulin treatment.

The plasma cf-DNA levels in the MARK-AGE and Health 2000 and the serum cf-DNA levels in the YFS were measured as previously described [17] using a QUANT-IT DNA High-Sensitivity Assay kit and a QUBIT Fluorometer (Invitrogen, Carlsbad, CA, USA) according to the manufacturer’s instructions.

The laboratory methods used to assess the biomarker levels are described in Online Resource 2.xlsx in Table S3–S5 and summarised in Online Resource 1.pdf, Table S6, S7, and S8. The blood and urinary biomarker data included a large number of biomolecules representing various biological domains (Table S7, S8, and S9 in Online Resource 1.pdf). Of the 142 biomarkers in the MARK-AGE, 24 were measured in urine and 118 in blood samples. In all cohorts, the largest proportion of the biomarkers consisted of metabolites that were measured using nuclear magnetic resonance spectroscopy.

### Statistical analysis

#### Age, sex, health behaviours and ageing phenotypes

First, the relationship of cf-DNA to age, sex, smoking, vegetable consumption, physical activity and ageing phenotypes (physical functioning, the number of diseases and frailty) were assessed one by one, using simple linear regression.

Next, to explore which of the relationships between cf-DNA and the above-mentioned variables are independent of each other, multivariate linear regression models were used. In model 1, we included age, sex, smoking, and vegetable consumption, and in model 2, physical activity was included in addition to the variables in model 1. In model 3, we included physical functioning and the number of diseases in addition to the variables in model 1. In model 4, we included frailty in addition to the variables in model 1. Due to the relatively low prevalence of chronic diseases in the youngest cohort (YFS), models 3 and 4 were fitted only in the two older cohorts (MARK-AGE and Health 2000). Scaled regression coefficients of the models in the different cohorts were summarised and visualised as forest plot using the R package jtools.

The *p*-value threshold was set to Bonferroni-adjusted *p*-value of 0.05 (= 0.05 was divided by the number of statistical tests).

In the statistical analysis, frailty index was used as a continuous variable, and the number of diseases was coded and used as 0, 1, 2 and 3 + diseases.

#### Biomarker analysis

In the analysis of the biomarker data, first, as a descriptive analysis, the associations between all biomarkers were explored using Spearman’s rank correlation coefficient statistics, and the correlation matrix was ordered using hierarchical clustering and visualised as a heatmap using the R-package ggcorrplot v0.1.3.

Then, associations of the cf-DNA levels with the biomarkers and metabolites were assessed using the Spearman correlation statistics. The *p*-value threshold was set to Bonferroni-adjusted *p*-value of 0.05 within each cohort (= 0.05 was divided by the number of statistical tests).

Biomarker profiles related to the cf-DNA levels were identified using a multivariate assessment based on a penalised regression method, Lasso [41] (available in the R package glmnet) and multivariate linear regression. The models were fitted separately in each cohort. All explanatory variables (the biomarkers) were standardised, and age and sex were included in the model. The Lasso is a feature selection method based on penalised least squares technique that imposes penalty to the sum of the absolute coefficients, shrinking the coefficients towards the null and minimising the risk of overfitting. That is, the Lasso selects those explanatory variables that are most strongly related to the dependent variable and shrinks weaker explanatory variables to 0. The Lasso is also robust against correlations between the explanatory variables. In the Lasso, the penalty parameter, λ with the minimum mean cross-validated error was selected using a tenfold cross-validation and used for the shrinkage of all the coefficients. As the result, the biomarkers that were not shrink to zero were ranked based on the (non-zero) coefficients. To select the final biomarker profile of the 30 top-ranking non-zero biomarkers in the Lasso and to obtain estimates and 95% confidence intervals (CIs) also for men and women, we further modelled these biomarkers using multivariate linear regression adjusted for age and sex. Of the 30 top-ranking non-zero biomarkers, those with a *p*-value < 0.05 in the linear regression were selected to the biomarker profile for the cf-DNA level. The profiles were visualised as forest plots using the R package jtools.

All statistical analyses and visualisations were performed using R software version 4.0.3.

## Results

### Relationships of cf-DNA levels to age, sex, health behaviours and ageing phenotypes

The characteristics of the analytical samples are shown in Table 1. First, we assessed, one by one, the relationship of cf-DNA to the following questionnaire and interview-based information: age, sex, health behaviours, (i.e., smoking, vegetable consumption, physical activity) and ageing phenotypes (i.e., physical functioning, number of diseases, and frailty). Results are shown in Online Resource 1.pdf in Table S10 (point estimates from the linear regression analysis). cf-DNA level was higher in men than in women in all cohorts (MARK-AGE: β =  − 0.0749, *p* = 1.02 × 10^−39^; YFS: β =  − 0.0587, *p* = 8.10 × 10^−11^; Health 2000: β =  − 0.0822, *p* = 6.40 × 10^−35^). Therefore, all main analyses were additionally stratified by sex.

**Fig. 1:**
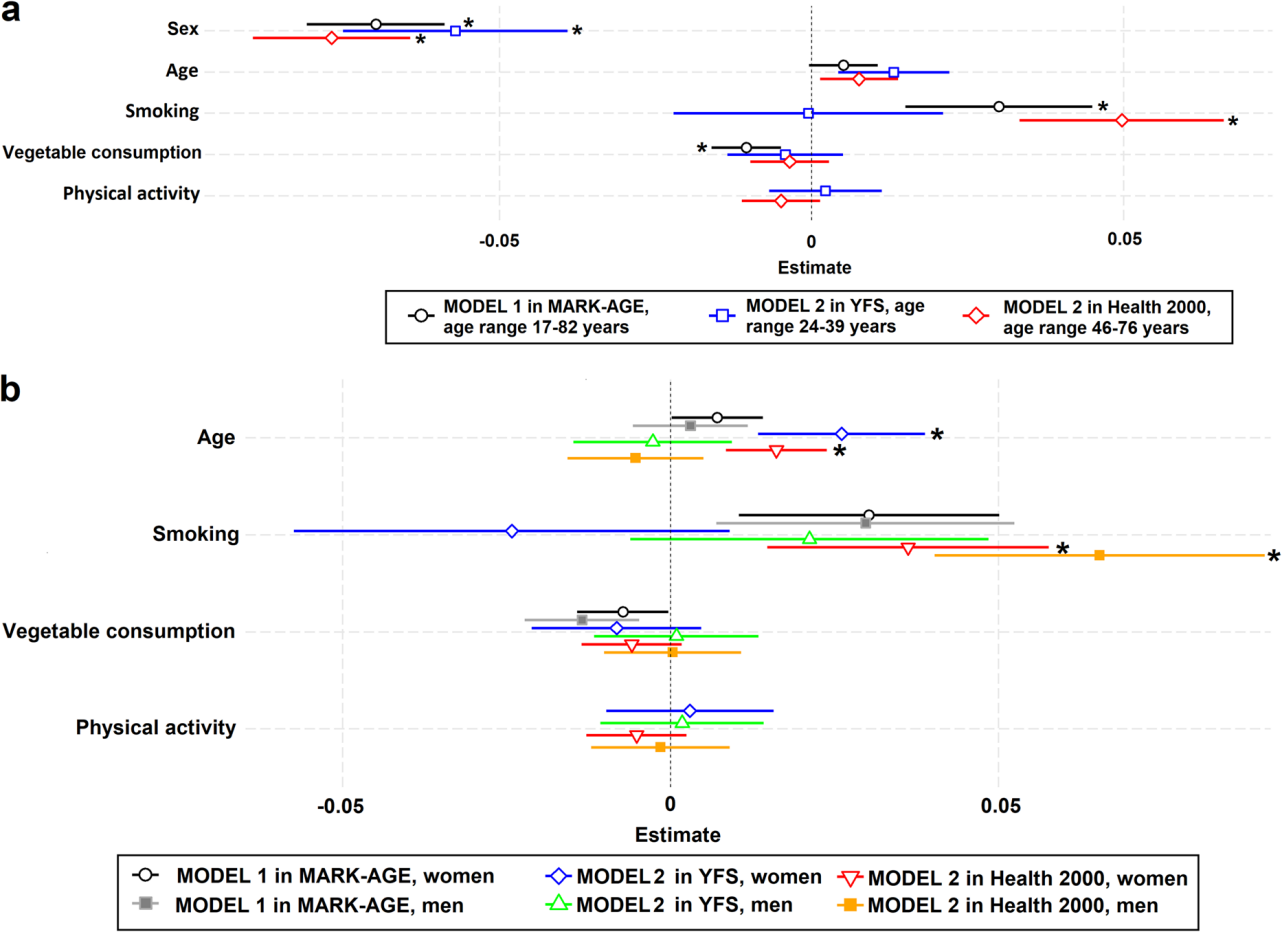
The relationship of cf-DNA levels to sex, age, smoking, vegetable consumption, and physical activity in the MARK-AGE, YFS and Health 2000. Results from multivariate linear regression models 1 and 2 are presented as forest plots in which regression coefficients and their unadjusted confidence intervals (95% CIs as whiskers) are shown for **a**) all participants in each cohort, and **b**) men and women separately. Due to data availability, Model 1 with sex, age, smoking, vegetable consumption but without physical activity was analysed in the MARK-AGE. Model 2 including also physical activity was analysed in the YFS and Health 2000. Bonferroni-adjusted *p*-values < 0.05 are indicated with *. The numeric point estimates for these models are shown in Table S11 in Online Resource 1.pdf

**Fig. 2:**
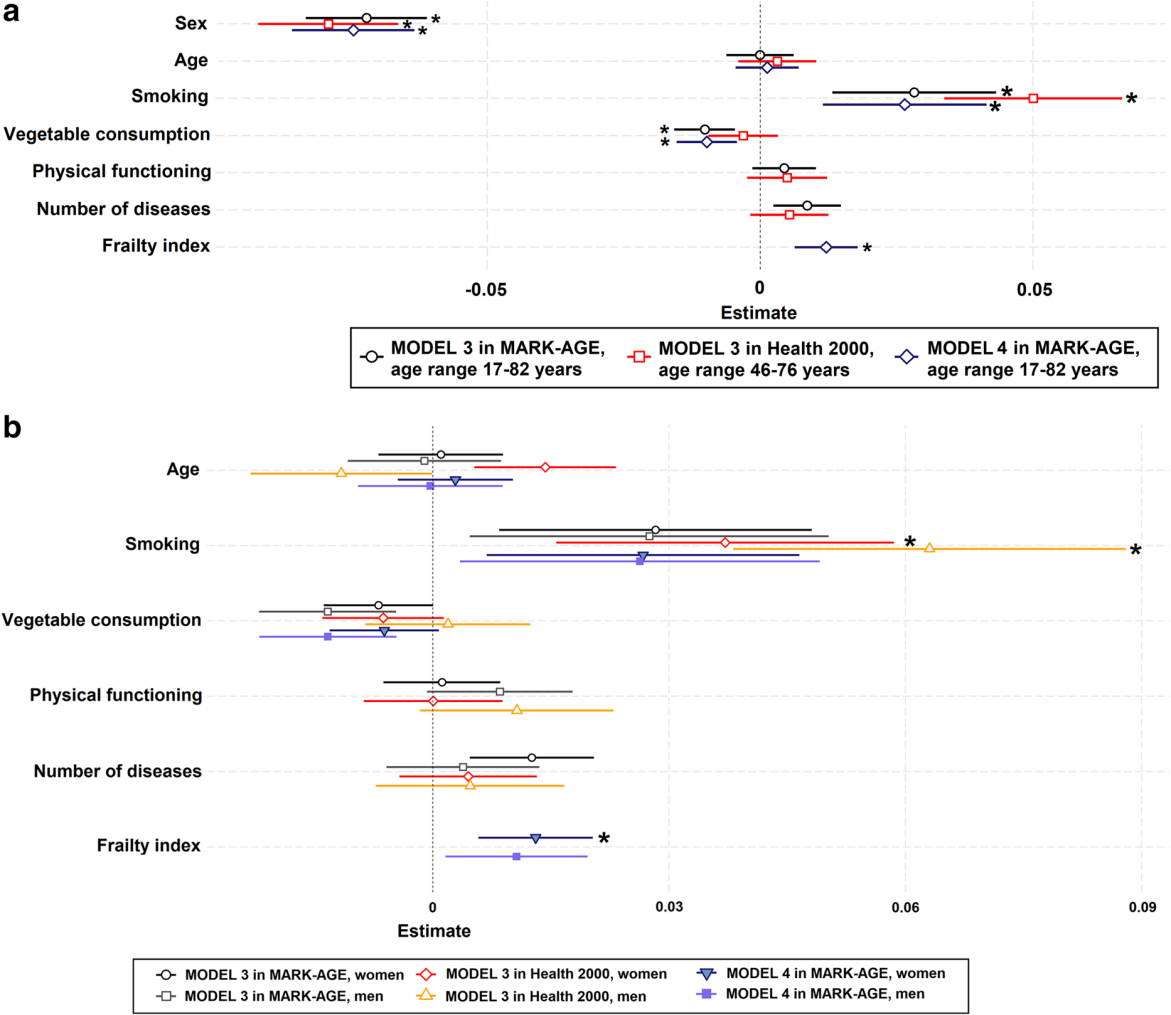
The relationship of cf-DNA levels to sex, age, smoking, vegetable consumption, physical functioning, the number of diseases, and frailty index in the Health 2000 and MARK-AGE. Results from multivariate linear regression models 3 and 4 are presented as forest plots in which regression coefficients and their unadjusted confidence intervals (95% CIs as whiskers) are shown **a**) for all participants in each cohort, and **b**) for men and women separately. Due to data availability, Model 3 with sex, age, smoking, vegetable consumption, physical functioning, the number of diseases but without frailty index was analysed in the MARK-AGE and Health 2000. Model 4 with sex, age, smoking, vegetable consumption and frailty index but without physical functioning and the number of diseases was analysed only in the MARK-AGE. Bonferroni-adjusted *p*-values < 0.05 are indicated with *. The numeric point estimates for these models are shown in Table S12 in Online Resource 1.pdf

After the simple linear regression analysis, we next explored using multivariate linear regression modelling which factors were related to cf-DNA levels adjusted for each other. Figure 1 (model 1 and 2) shows the relationship to age, sex, and health behaviours (smoking, vegetable consumption, physical activity), and Fig. 2 (model 3 and 4) relationship to age, sex, health behaviour (smoking, vegetable consumption), and ageing phenotypes (physical functioning, the number of diseases, and frailty), also stratified by sex. The corresponding standardised regression coefficients and *p*-values are shown in Table S11 and S12 (Online Resource 1.pdf).

**Fig. 3:**
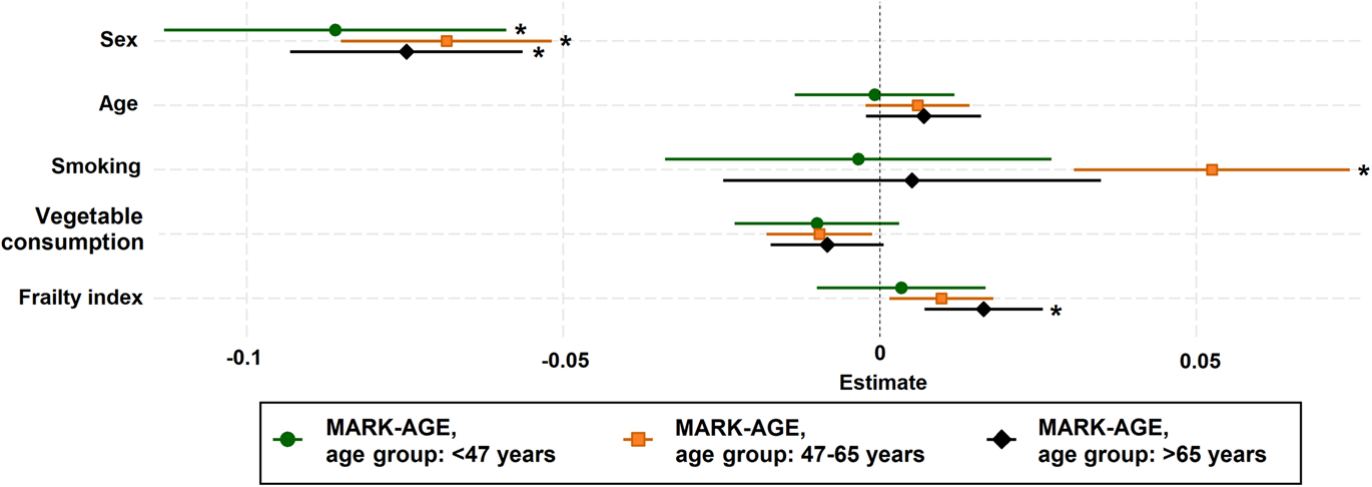
The relationship of cf-DNA levels to sex, age, smoking, vegetable consumption, and frailty index (model 4) in three age groups (< 47, 47–65, > 65 years) in the MARK-AGE. Results from the age group-stratified sensitivity analysis are presented as a forest plot that shows regression coefficients and their unadjusted confidence intervals (95% CIs as whiskers). Bonferroni-adjusted *p*-values < 0.05 is indicated with *. Numeric point estimates of this multivariate assessment are shown in Table S13 in Online Resource 1.pdf

In all cohorts and models, cf-DNA level was significantly higher in men compared to women after adjusting for the other variables. In model 1 and 2 (Fig. 1), a higher cf-DNA level was related to a higher age in women in the YFS and the Health 2000. cf-DNA level was also significantly higher in smokers in the full analytical sample of the MARK-AGE (Fig. 1: model 1, Fig. 2: model 3 and 4), and in the Health 2000 in both men and women (Fig. 1: model 1 and 2, Fig. 2: model 3), but not in the YFS (Fig. 1: model 1 and 2). A lower cf-DNA level was related to a higher vegetable consumption in the full analytical sample of the MARK-AGE (Fig. 1: model 1, Fig. 2: model 3 and 4), but not in the other cohorts. cf-DNA was not related to physical activity, physical functioning, or the number of diseases (Fig. 1 and 2: model 1–3). In model 4 in the MARK-AGE, a higher cf-DNA level was related to a higher degree of frailty in the full analytical sample and in women. As a sensitivity analysis, the model 4 was analysed stratified by age (Fig. 3, and Online Resource 1.pdf: Table S13). cf-DNA level was higher in men in all three age groups (< 47, 47–65, > 65 years), and in the 47–65-year-old smokers and in more frail individuals older than 65 years.

### Biomarkers related to the cf-DNA levels

The distributions of the biomarkers in the analysis and Spearman correlations statistics for each biomarker with the cf-DNA level are presented in Table S3–S5 in Online Resource 2.xlsx. In the MARK-AGE, 142 blood and urine biomarkers in 1479 individuals (826 women and 648 men), in the YFS, 147 blood biomarkers in 1701 individuals (931 women and 770 men), and in the Health 2000, 241 blood biomarkers in 1196 individuals (707 women and 489 men) were available for all individuals (complete cases) and thus included in the analysis. Correlation matrices across the biomarkers are presented as heatmaps in Fig. S1, S2 and S3 in the Online Resource 3.7z. In total, 57 (40%) biomarkers in the MARK-AGE (50 measured in blood and 7 in urine), 64 (44%) in the YFS, and 26 (11%) in the Health 2000, were statistically significantly associated with the cf-DNA levels in the full analytical samples after correction for multiple testing (Bonferroni-adjusted *p*-value < 0.05). Of these biomarkers, the ones that were associated with cf-DNA after correction for multiple testing in both men and women are shown in Table 2. The directions of these associations were similar in men and women (Table 2). The strongest biomarker correlate for cf-DNA was the proportion of red blood cells, *r* = 0.35, *p* = 4 × 10^−27^ (Table 2).

**Table 2:** Biomarkers associated with cf-DNA levels in the correlation analysis in the full sample, men and women in the (A) MARK-AGE, (B) YFS and (C) Health 2000. The table shows the Spearman correlation coefficients (*r*) and unadjusted *p*-values for those biomarkers that were associated with the cf-DNA level in both men and women after correction for multiple testing (Bonferroni-adjusted *p*-value < 0.05)

			All		Women		Men	
Sample	Domain	Biomarker	*r*	*p*	*r*	*p*	*r*	*p*
**A**								
MARK-AGE	Oxygen transfer	Proportion of red blood cells in blood	0.352	3.85 × 10^−27^	0.205	9.36 × 10^−05^	0.185	1.80 × 10^−06^
	One carbon metabolism	Plasma Homocysteine	0.280	1.73 × 10^−22^	0.190	4.97 × 10^−07^	0.182	1.52 × 10^−05^
	Protein modification	Relative amount of peak 9 N-glycan on total serum proteins	0.227	1.42 × 10^−15^	0.132	3.40 × 10^−04^	0.156	1.55 × 10^−04^
	Immune system	Plasma C-reactive protein	0.166	6.76 × 10^−09^	0.145	1.21 × 10^−05^	0.211	9.65 × 10^−05^
	Nutrition	Plasma carotenoid lutein	− 0.199	1.07 × 10^−11^	− 0.177	1.39 × 10^−04^	− 0.179	1.03 × 10^−05^
	Nutrition	Plasma ascorbic acid	− 0.244	3.66 × 10^−15^	− 0.176	1.82 × 10^−04^	− 0.208	9.91 × 10^−07^
**B**								
YFS	Low-molecular-weight metabolites	3-hydroxybutyrate	0.230	8.63 × 10^−17^	0.256	2.43 × 10^−13^	0.244	5.21 × 10^−07^
	Lipid extract metabolites	Ratio of bisallylic groups to total fatty acids	0.197	1.62 × 10^−16^	0.285	1.13 × 10^−17^	0.151	1.51 × 10^−05^
	Lipid extract metabolites	Ratio of bisallylic groups to double bonds	0.196	6.84 × 10^−17^	0.274	3.59 × 10^−17^	0.154	1.50 × 10^−05^
	Lipid extract metabolites	Average number of double bonds in a fatty acid chain	0.172	1.28 × 10^−12^	0.255	1.95 × 10^−13^	0.130	6.40 × 10^−05^
	Lipid extract metabolites	Description of average fatty acid chain length	0.156	3.93 × 10^−11^	0.225	5.71 × 10^−10^	0.116	7.16 × 10^−05^
	Lipoprotein subclasses	Cholesterol esters in medium VLDL	− 0.075	2.31 × 10^−04^	− 0.119	1.70 × 10^−04^	− 0.128	3.76 × 10^−05^
	Lipoprotein subclasses	Triglycerides in chylomicrons and extremely large VLDL	− 0.078	1.23 × 10^−04^	− 0.157	1.18 × 10^−06^	− 0.110	1.03 × 10^−04^
	Lipoprotein subclasses	Total lipids in small VLDL	− 0.080	3.35 × 10^−04^	− 0.144	4.93 × 10^−05^	− 0.139	2.87 × 10^−05^
	Lipoprotein subclasses	Total lipids in chylomicrons and extremely large VLDL	− 0.084	7.74 × 10^−05^	− 0.159	9.93 × 10^−07^	− 0.118	6.70 × 10^−05^
	Lipoprotein subclasses	Phospholipids in small VLDL	− 0.084	1.94 × 10^−04^	− 0.151	3.10 × 10^−05^	− 0.130	8.26 × 10^−05^
	Lipoprotein subclasses	Concentration of small VLDL particles	− 0.091	6.76 × 10^−05^	− 0.155	1.60 × 10^−05^	− 0.154	3.03 × 10^−06^
	Lipoprotein subclasses	Concentration of chylomicrons and extremely large VLDL particles	− 0.094	2.29 × 10^−05^	− 0.126	1.54 × 10^−05^	− 0.124	4.86 × 10^−05^
	Lipoprotein subclasses	Concentration of very large VLDL particles	− 0.094	1.18 × 10^−05^	− 0.187	2.35 × 10^−08^	− 0.119	4.42 × 10^−05^
	Lipoprotein subclasses	Total cholesterol in medium VLDL	− 0.097	1.19 × 10^−05^	− 0.155	3.41 × 10^−06^	− 0.144	6.12 × 10^−06^
	Lipoprotein subclasses	Phospholipids in very large VLDL	− 0.108	3.91 × 10^−06^	− 0.202	1.13 × 10^−08^	− 0.121	3.65 × 10^−05^
	Lipoprotein subclasses	Triglycerides in small VLDL	− 0.108	5.76 × 10^−06^	− 0.170	3.80 × 10^−06^	− 0.178	6.03 × 10^−08^
	Lipoprotein subclasses	Total lipids in very large VLDL	− 0.108	2.36 × 10^−06^	− 0.205	5.02 × 10^−09^	− 0.125	1.94 × 10^−05^
	Lipoprotein subclasses	Phospholipids in chylomicrons and extremely large VLDL	− 0.112	9.98 × 10^−06^	− 0.196	1.67 × 10^−08^	− 0.117	1.34 × 10^−04^
	Lipoprotein subclasses	Cholesterol esters in large VLDL	− 0.112	1.20 × 10^−06^	− 0.181	2.67 × 10^−07^	− 0.166	1.36 × 10^−07^
	Lipoprotein subclasses	Triglycerides in very large VLDL	− 0.113	1.68 × 10^−06^	− 0.212	2.24 × 10^−09^	− 0.129	1.81 × 10^−05^
	Lipids	Serum total triglycerides	− 0.114	1.51 × 10^−06^	− 0.169	1.95 × 10^−06^	− 0.165	7.44 × 10^−07^
	Lipoprotein subclasses	Triglycerides in VLDL (Lipido)	− 0.116	1.24 × 10^−06^	− 0.170	1.94 × 10^−06^	− 0.177	1.28 × 10^−07^
	Lipoprotein subclasses	Phospholipids in medium VLDL	− 0.116	1.18 × 10^−06^	− 0.174	6.13 × 10^−07^	− 0.170	2.76 × 10^−07^
	Lipoprotein subclasses	Total lipids in medium VLDL	− 0.117	1.14 × 10^−06^	− 0.178	3.37 × 10^−07^	− 0.171	2.78 × 10^−07^
	Lipoprotein subclasses	Mean diameter for VLDL particles	− 0.117	6.71 × 10^−07^	− 0.189	4.55 × 10^−08^	− 0.169	1.94 × 10^−07^
	Lipoprotein subclasses	Concentration of medium VLDL particles	− 0.119	8.16 × 10^−07^	− 0.180	2.45 × 10^−07^	− 0.175	1.82 × 10^−07^
	Lipoprotein subclasses	Triglycerides in VLDL	− 0.120	7.73 × 10^−07^	-0.186	1.56 × 10^−07^	-0.176	1.61 × 10^−07^
	Lipoprotein subclasses	Triglycerides in medium VLDL	− 0.122	6.23 × 10^−07^	− 0.182	1.61 × 10^−07^	− 0.181	1.12 × 10^−07^
	Lipoprotein subclasses	Free cholesterol in medium VLDL	− 0.123	2.93 × 10^−07^	− 0.197	2.35 × 10^−08^	− 0.161	1.10 × 10^−06^
	Lipoprotein subclasses	Total cholesterol in large VLDL	− 0.123	3.21 × 10^−07^	− 0.205	9.70 × 10^−09^	− 0.158	6.95 × 10^−07^
	Lipoprotein subclasses	Concentration of large VLDL particles	− 0.129	1.53 × 10^−07^	− 0.206	5.16 × 10^−09^	− 0.161	1.01 × 10^−06^
	Lipid extract metabolites	Total triglycerides	− 0.129	1.43 × 10^−07^	− 0.198	1.05 × 10^−08^	− 0.138	5.52 × 10^−05^
	Lipoprotein subclasses	Free cholesterol in large VLDL	− 0.130	1.29 × 10^−07^	− 0.222	5.75 × 10^−10^	− 0.148	3.72 × 10^−06^
	Lipoprotein subclasses	Phospholipids in large VLDL	− 0.130	1.73 × 10^−07^	− 0.199	1.11 × 10^−08^	− 0.168	6.85 × 10^−07^
	Lipoprotein subclasses	Total lipids in large VLDL	− 0.131	1.71 × 10^−07^	− 0.209	5.28 × 10^−09^	− 0.166	6.21 × 10^−07^
	Lipoprotein subclasses	Triglycerides in large VLDL	− 0.131	1.75 × 10^−07^	− 0.207	5.32 × 10^−09^	− 0.166	6.77 × 10^−07^
	Low-molecular-weight metabolites	CH2 groups of mobile lipids	− 0.142	4.81 × 10^−09^	− 0.219	1.49 × 10^−10^	− 0.126	9.72 × 10^−05^
	Lipids	Triglycerides	− 0.149	9.96 × 10^−08^	− 0.203	3.09 × 10^−09^	− 0.184	4.33 × 10^−06^
Abbreviations: *Lipido*, computationally estimated measures, *YFS*,Young Finns Study, *VLDL*, very low density lipoprotein								
**C**								
Health 2000	Inflammation	Interleukin 6	0.202	1.24 × 10^−09^	0.179	7.33 × 10^−05^	0.197	2.84 × 10^−06^
	Low-molecular-weight metabolites	3-hydroxybutyrate	0.184	5.46 × 10^−13^	0.184	5.84 × 10^−05^	0.123	1.02 × 10^−07^
	Inflammation	C-reactive protein	0.169	4.27 × 10^−09^	0.175	2.20 × 10^−05^	0.177	8.66 × 10^−07^

**Fig. 4:**
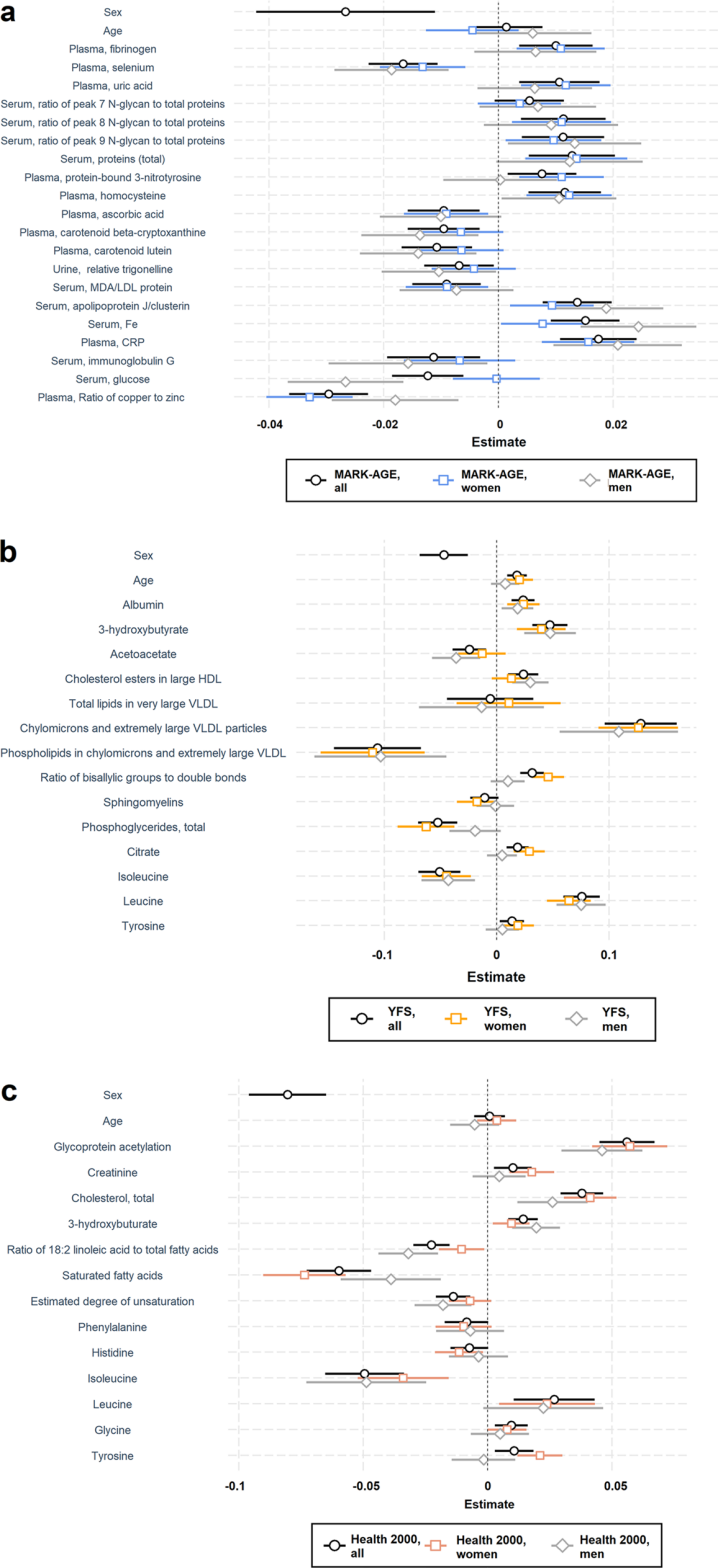
Biomarker profiles related to cf-DNA levels. The biomarkers in blood and urine were identified first using a feature selection assessment, the Lasso after which the top-ranking biomarkers were modelled in multivariate linear regression. The final model was considered as the cf-DNA-related biomarker profile. The profiles are presented as forest plots in which regression coefficients and their unadjusted confidence intervals (95% CIs as whiskers) are shown for **a**) MARK-AGE, **b**) YFS, and **c**) Health 2000, and stratified by sex. Point estimates of the final multivariate models are shown in Table S14 in Online Resource 1.pdf. Abbreviations: CRP = C-reactive protein, HDL = high density lipoprotein, LDL = low density lipoprotein, MDA = malondialdehyde, VLDL = very low density lipoprotein, * = eluting with retention time of Albumin or Selenoprotein P

After the correlation analysis, we next explored which biomarkers were related to the cf-DNA levels adjusted for each other using the penalised Lasso. The number of non-zero coefficients in the Lasso was 51 in MARK-AGE, 64 in YFS, and 53 in Health 2000 (Online Resource 2.xlsx: Table S3, S4, S5). Then, the biomarkers to be included in the biomarker profiles were selected of the top-ranking non-zero biomarkers using multivariate linear regression. The biomarkers profiles, coefficients and 95% CIs, also stratified by sex, are shown in Fig. 4a–c and in Online Resource 1.pdf in Table S14.

As a sensitivity analysis in the biomarker data, the correlation analysis was performed additionally in 985 non-smokers (after excluding 211 [21%] smokers of the 1196 individuals) in the Health 2000 data. The results in the non-smoking participants (all: n = 985; women: n = 606, men: n = 379) are shown in Table S15 (Online Resource 2.xlsx). When men and women were analysed together, 29 (12%) of the 240 biomarkers were significant correlates for cf-DNA, and of those, CRP and 3-hydroxybutyrate were correlates that were consistent in men and women. The biomarker profile identified in the full analytical sample of the Health 2000 (Fig. 4c), was also analysed in the non-smokers (Online Resource 1.pdf: Fig. S4).

## Discussion

In this study, the relationship of circulating cf-DNA levels to age, sex, health behaviours (smoking, physical activity, vegetable consumption), ageing phenotypes (physical functioning, the number of diseases, frailty) and an extensive panel of biomarkers, including metabolomics, measured in blood and urine were assessed. The analysis was performed in three cohorts, comprising 5385 individuals with an age range from 17 to 82 years. As cf-DNA has emerged as a viable biomarker of ageing and tissue damage, showing utility in risk stratification in various conditions (see e.g., references 6–15), and information on phenotypes and metabolic processes underlying the cf-DNA levels is lagging behind, a catalogue of associations with a range of health attributes and conditions is needed. Moreover, the biomarker associations can inform us about possible underlying biological processes that can lead to elevated cf-DNA levels. Our results show that the cf-DNA level was higher in men in all cohorts with varying age ranges, and especially, in middle-aged men and women who smoke, and in older more frail individuals. In the biomarker analysis, a focus was set to the similarities in the associations in men and women, and the associations were consistent. Correlation statistics of the biomarker data showed that the cf-DNA level associated with low-grade inflammation (CRP, IL-6), higher levels of homocysteine, higher proportion of red blood cells and lower levels of ascorbic acid. Inflammation (reflected by elevated levels of CRP and glycoprotein acetylation, GlycA), amino acids (isoleucine, leucine, tyrosine), and ketogenesis (3-hydroxybutyrate) emerged into the cf-DNA level-related biomarker profiles in at least two of the cohorts. The overlap of the biomarkers available for analysis across the cohorts was limited and therefore, we performed a data-driven analysis rather than followed conventional analysis approaches such as discovery and replication analyses. However, although the biomarker availability varied and the cohorts comprised differing population characteristics such as age range, we observed similar associations in the different cohorts and among the self-reported and biomarker data.

The sex and age-associations with cf-DNA have been studied previously, and the results have been relatively inconsistent [20, 42]. In our analysis, cf-DNA was not related to chronological age consistently across the samples, whereas sex was; men had evidently higher cf-DNA levels in all our analyses. In the previous analyses, sample sizes have been smaller, and usually a limited number of health factors have been considered. However, in accordance with our observation on the sex difference, for example, Meddeb et al. (2019) reported that healthy men have significantly higher levels of both nuclear and mitochondrial circulating DNA compared to women [42]. As men and women differ in their body composition [43], we hypothesise that e.g. higher muscle mass and red blood cell count [44] and faster metabolic turnover in men [45] might lead to higher cellular release of cf-DNA and partly explain the sex difference in healthy humans. This matter is however yet to be studied.

We observed that the cf-DNA level was especially higher in middle-aged smokers in the Health 2000 and MARK-AGE compared to middle-aged non-smokers. To our knowledge, research evidence on circulating cf-DNA association with smoking is scarce, and the findings have been inconsistent [20], and the mechanisms or the pathways involved in the smoking-associated changes in the cf-DNA levels are not fully characterised. Smoking alters and activates inflammatory pathways [46] and compromises vascular health [47], and the influence of cigarette smoking is seen particularly on the endothelial cells. Furthermore, Hayun, Shoham et al. (2019) have reported that smoke inhalation in the event of fire induces temporal increases in cf-DNA levels [48]. We hypothesise that vascular health and inflammatory pathways might underlie the association between cf-DNA and smoking.

Frailty index is a multidimensional indicator of ageing-related accumulation of health deficits, and a strong predictor of mortality [49, 50]. The Rockwood frailty index [51, 52] covers not only morbidity and physiological functioning but also symptoms, self-rated health, and psychological aspects. The items for the index were available for our analysis only in the MARK-AGE. Even after adjusting for age, sex and health behaviours, frailty remained related to a higher cf-DNA level. The age group-stratified sensitivity analysis showed that the relationship was the strongest in individuals who were older than 65 years and women. This is in accordance with our previous findings in which total concentration of cf-DNA as well as the levels of different cf-DNA species were higher in more frail older individuals [16].

Vegetable consumption, physical activity, physical functioning, and the number of diseases showed less consistent results across the samples or were not significant after the analysis was adjusted for the other health factors. Higher self-reported vegetable consumption was associated with a lower cf-DNA level in the Health 2000 and MARK-AGE in the unadjusted analysis, and when adjusted for the other health factors, the relationship remained significant only in the MARK-AGE. The number of diseases was higher with increased cf-DNA level in women in the MARK-AGE in the simple linear regression analysis only. Neither physical activity, nor physical functioning was related to the cf-DNA levels.

In the biomarker data, we first explored the correlates for cf-DNA that are common for men and women at single-biomarker-level using correlation statistics. Then, using feature selection methodology (penalised regression) and linear regression, we built biomarker profiles associated with cf-DNA levels in all three cohorts. Our findings in biomarker data were in line with earlier reports and new biomarkers associated with cf-DNA were identified. As a sensitivity analysis, the biomarker profile in the Health 2000 was analysed also in non-smokers and the results were consistent. This suggests that the biomarker profiles were not driven by the smoking status. In the two older cohorts (MARK-AGE and Health 2000), a higher inflammation level was associated with a higher cf-DNA level. In MARK-AGE, CRP was a positive correlate for cf-DNA in the unadjusted analysis and a feature that reflects inflammation in the biomarker profile. Previously, low-grade inflammation reflected by a higher CRP level was associated with higher cf-DNA levels in middle-aged and older individuals [16, 17]. In the biomarker profile in the Health 2000, glycoprotein acetylation (GlycA) [53] represented inflammation. GlycA levels were higher with higher levels of cf-DNA in the profile, and this relationship was similar regardless of participants’ sex or smoking status. Earlier studies have shown that increased blood levels of GlycA predicts morbidity [54] and mortality [55, 56], reflects gut microbiome diversity [57] and associates with alcohol consumption [58]. In the younger cohort (YFS), biomarkers of inflammation were not associated with cf-DNA. As inflammation increases with advancing age, these observations were as expected.

The identified biomarker profiles showed some differences across the cohorts, partly owing to the fact that the available biomarker data varied by the cohorts. Nevertheless, a consistent and new finding in the youngest (YFS) and the middle-aged cohort (Health 2000) was that the cf-DNA-related biomarker profile included 3-hydroxybutyrate, leucine, tyrosine and isoleucine. Three-hydroxybutyrate, leucine and tyrosine levels were higher and isoleucine levels lower with higher cf-DNA levels. Fasting, uncontrolled type 1 diabetes, increased energy demand by exercise, and ketogenic diet all induce an accumulation of circulating ketones [59–61], and may be an underlying factor for variation in 3-hydroxybutyrate. Three-hydroxybutyrate is considered as a promising target for deceleration of the ageing process, as increased levels of 3-hydroxybutyrate by e.g., dietary modulation are linked to decreased cellular ageing, suppression of inflammation and senescence, and improvement in metabolic homeostasis and neural regeneration [62]. Tyrosine is a non-essential amino acid that can be either gluconeogenic or ketogenic and is used in the melatonin synthesis [63]. Tyrosine level associates with mortality in patients with coronary artery disease [64], and alcohol consumption [58]. Tyrosine metabolism is one of the metabolic pathways included in the metabolic age predictor that constitute a very recent biological age algorithm [65]. Leucine and isoleucine are essential, branched-chain amino acids (BCAAs) and ketogenic, while isoleucine can also be glucogenic [66]. Leucine upregulates protein synthesis through mTOR pathway [67]. High blood levels of BCAAs and tyrosine are also associated with the risk of developing type 2 diabetes [68, 69], and BCAAs are also considered as biomarkers of cardiovascular health [70]. As the roles of these amino acids in health and disease are complex, it is possible that their relationship to the cf-DNA levels reflects normal variation in metabolic processes or indicates a state of an increased risk of pathologies.

Unfavourable lipid metabolism profile of major lipid and lipoprotein cholesterol fractions in the blood (lower levels of HDL cholesterol, for instance) has already been associated with increased cf-DNA levels in the Health 2000 [17]. Our current analysis was extended to include a more comprehensive metabolomics data. As a new finding, we found that in the biomarker profile in the Health 2000, higher ratio of 18:2 linoleic acid (LA) to total fatty acids was related to lower cf-DNA-levels. Polyunsaturated LA is a precursor for the other omega-6 fatty acids and considered as anti-atherogenic [71]. Human body cannot synthesise it and primary dietary sources of LA are vegetable oils and nuts. In the youngest cohort (YFS), but not in the older cohort (Health 2000, where also available for analysis), correlation analysis revealed higher levels of various VLDL subclasses associated with lower cf-DNA levels. In the biomarker profile in the YFS, higher level of chylomicrons and extremely large VLDL particles and lower levels of phospholipids in chylomicrons and extremely large VLDL were related to increased cf-DNA levels. Overproduction of VLDLs, in general, is a health risk, a sign of dyslipidaemia [72]. Previous studies indicate that VLDL particle size is associated with a higher alcohol consumption [58], and mortality [55, 56]. Phospholipid composition of the lipoproteins differs between men and (non-pregnant) women [73], but not between obese and lean pregnant women [74]. In an analysis where the lipoprotein phospholipids were not separated from the different lipoproteins, phospholipids were associated with obesity and insulin resistance in young adults [75]. Although higher cf-DNA levels seem to robustly associate with an unfavourable lipid profile, further analyses are needed to shed light into the mechanisms underlying our findings on the different VLDL species.

The representation of the biomarker domains available for analysis was the richest in the MARK-AGE when compared to the YFS and the Health 2000. That is, many biomarkers, such as plasma β-cryptoxanthin and lutein and the proportion of the red blood cells were available for analysis only in the MARK-AGE data. As discussed above, we observed in MARK-AGE that a higher self-reported vegetable consumption is linked to lower cf-DNA levels. In line with this, in the cf-DNA-related biomarker profile in the MARK-AGE, cf-DNA level was lower with higher levels of biomolecules that are considered as indications of diet rich in vegetables and fruits (through e.g. elevated levels of plasma β-cryptoxanthin and lutein [76]). Therefore, our results suggest that diet might be a modulator of the cf-DNA levels. In the correlation analysis, the highest-ranking correlate for cf-DNA in MARK-AGE data was the proportion of the red blood cells. The higher levels of cf-DNA, the higher is the proportion of these cells as shown in our analysis and elsewhere [3]. A possible underlying pathway for this association might be enucleation during the erythrocyte maturation [77]. In this process, the erythroblasts expel their nuclei to be degraded. The higher the erythrocyte count, the higher the amount of secreted chromatin. However, the amount of cf-DNA released into the circulation by erythrocyte enucleation is still unclear. As the last example of the many findings in our analysis, homocysteine level in blood was a positive correlate for cf-DNA in men and women in the unadjusted analysis and a feature in the cf-DNA-related biomarker profile in the MARK-AGE. However, in the youngest cohort (YFS) where it was also analysed, cf-DNA level was not related to homocysteine level. Homocysteine is an indicator of e.g. worse cardiovascular health [78], an important component in the one carbon cycle that supports multiple cellular processes, and included in a metabolic signature that mediates longevity-related effects in different species [79]. Thus, our findings suggest that a higher cf-DNA level correlates with metabolic signs of poorer health.

When interpreting our findings, some issues should be considered. First, as this analysis was performed in cross-sectional data sets, analyses in longitudinal settings are needed to clarify causal relationships within cf-DNA level-modifying pathways. Second, in the YFS, the general level of cf-DNA was higher than in the other cohorts. In the YFS, cf-DNA was measured in serum and in others in plasma. Thus, a potential explanation for the difference may be the sample type because previous comparative analyses have shown cf-DNA level is higher in serum than in plasma [80–82]. Lastly, as our quantification method did not allow determination of the cf-DNA fragment size, we are unable to discern whether some of the inter-individual differences in the cf-DNA levels arise from nuclease activity and/or individual clearance rates.

In conclusion, in this explorative analysis in three large human cohorts, we found that the circulating cf-DNA level associates with various health factors including risk factors and signs of health decline measured by subjective (questionnaire and interview) and objective (blood and urinary biomarkers including metabolomics) assessments. The fact that higher cf-DNA levels clustered with known metabolic and cardiovascular risk factors suggests that it might index the cardiometabolic risk. These results provide essential information for future studies in which biological pathways of the cf-DNA are analysed as well as for studies assessing the cf-DNA level as a clinical marker.

## Supplementary Information

Below is the link to the electronic supplementary material.

Online resource 1 (PDF 453 KB)

Online resource 2 (XLSX 168 KB)

Online resource 3 (7Z 7721 KB)

## Data Availability

The data used in the current study cannot be stored in public repositories or otherwise made publicly available due to ethical restrictions. However, data are available upon request from the MARK-AGE, YFS, and Health 2000 survey for researchers who meet the criteria for access to confidential data. Data from the MARK-AGE study are available from the MARK-AGE steering committee (contact: Professor Alexander Bürkle, alexander.buerkle@uni-konstanz.de). Regarding YFS, investigators can submit an expression of interest to the chairman of the data sharing and publication committee (Professor Mika Kähönen, Tampere University, Finland). The Health 2000 data are available from THL on request, subject to the submission of approved study proposals and a data transfer agreement (contact: terveys-2000–2011@thl.fi).
