# Supplementary material for: Circulating cell-free DNA in health and disease — the relationship to health behaviours, ageing phenotypes and metabolomics
Source: GeroScience. 2022 Jul 21;45(1):85–103. doi: 10.1007/s11357-022-00590-8 (PMC9886738; doi:10.1007/s11357-022-00590-8)
Supplement: Supplementary file 1 — Online resource 1 (PDF 453 KB) [file 11357_2022_590_MOESM1_ESM.pdf]

Circulating cell-free DNA in health and disease --- the relationship to health behaviours, ageing phenotypes and metabolomics

Laura Kananen[1,2,3]*, Mikko Hurme[3], Alexander Bürkle[4], Maria Moreno-Villanueva[4], Jürgen Bernhardt[5], Florence Debacq-Chainiaux[6], Beatrix Grubeck-Loebenstein[7], Marco Malavolta[8], Andrea Basso[8], Francesco Piacenza[8], Sebastiano Collino[9], Efstathios S Gonos[10], Ewa Sikora[11], Daniela Gradinaru[12], Eugene HJM Jansen[13], Martijn ET Dollé[13], Michel Salmon[14], Wolfgang Stuetz[15], Daniela Weber[16], Tilman Grune[16,17,18], Nicolle Breusing[18], Andreas Simm[19], Miriam Capri[20], Claudio Franceschi[20], Eline Slagboom[21], Duncan Talbot[22], Claude Libert[23,24], Jani Raitanen[2], Seppo Koskinen[25], Tommi Härkänen[25], Sari Stenholm[26,27], Mika Ala-Korpela[28], Terho Lehtimäki[29], Olli T Raitakari[30], Olavi Ukkola[31], Mika Kähönen[32], Marja Jylhä[2], Juulia Jylhävä[1,2]

[1] Department of Medical Epidemiology and Biostatistics, Karolinska Institutet, Stockholm, Sweden

[2] Faculty of Social Sciences (Health Sciences), and Gerontology Research Center, Tampere University, Tampere, Finland

[3] Faculty of Medicine and Health Technology, and Gerontology Research Center, Tampere University, Tampere, Finland

[4] Molecular Toxicology Group, University of Konstanz, Germany

[5] BioTeSys GmbH, 73728 Esslingen, Germany

[6] University of Namur, URBC-Narilis, Rue de Bruxelles, 61, Namur B-5000, Belgium

[7] Research Institute for Biomedical Aging Research, University of Innsbruck, Rennweg, 10, 6020 Innsbruck, Austria

[8] Advanced Technology Center for Aging Research, Scientific Technological Area, IRCCS INRCA, Ancona, Italy

[9] Nestlé Research, Nestlé Institute of Health Sciences, EPFL Innovation Park 1015 Lausanne, Switzerland

[10] National Hellenic Research Foundation, Institute of Biology, Medicinal Chemistry and Biotechnology, Athens, Greece

[11] Laboratory of the Molecular Bases of Ageing, Nencki Institute of Experimental Biology, Polish Academy of Sciences, 3 Pasteur street, 02-093 Warsaw, Poland

[12] Department of Biochemistry, Faculty of Pharmacy, “Carol Davila” University of Medicine and Pharmacy, 020956 Bucharest, Romania

[13] National Institute for Public Health and the Environment (RIVM), Centre for Health Protection, P.O. Box 1, 3720 BA Bilthoven, The Netherlands

[14] Straticell, Science Park Crealys, Rue Jean Sonet 10, 5032 Les Isnes, Belgique

[15] Institute of Nutritional Sciences (140), University of Hohenheim, 70593 Stuttgart, Germany

[16] Department of Molecular Toxicology, German Institute of Human Nutrition Potsdam-Rehbruecke (DIfE), Nuthetal, Germany

[17] Department of Physiological Chemistry, Faculty of Chemistry, University of Vienna, 1090 Vienna, Austria

[18] Institute of Nutritional Medicine (180), University of Hohenheim, 70593 Stuttgart, Germany

[19] Department of Cardiothoracic Surgery, University Hospital Halle, Ernst-Grube Str. 40, D-06120 Halle (Saale), Germany

[20] DIMES- Department of Experimental, Diagnostic and Specialty Medicine; Interdepartmental Center "Alma Mater Research Institute on Global Challenges and Climate Change (Alma Climate)", ALMA MATER STUDIORUM, University of Bologna, 40126 Bologna, Italy

[21] Section of Molecular Epidemiology, Leiden University Medical Centre, Leiden, The Netherlands

[22] Unilever Science and Technology, Beauty and Personal Care, Sharnbrook, United Kingdom

[23] Center for Inflammation Research, VIB, Ghent, Belgium

[24] Department of Biomedical Molecular Biology, Ghent University, Ghent, Belgium

[25] National Institute for Health and Welfare, Finland

[26] Department of Public Health, University of Turku and Turku University Hospital, Turku, Finland

[27] Centre for Population Health Research, University of Turku and Turku University Hospital; Turku, Finland

[28] Computational Medicine, Faculty of Medicine, University of Oulu and Biocenter Oulu, Oulu, Finland; Center for Life Course Health Research, University of Oulu, Oulu, Finland; NMR Metabolomics Laboratory, School of Pharmacy, University of Eastern Finland, Kuopio, Finland

[29] Faculty of Medicine and Health Technology, Tampere University, and Finnish Cardiovascular Research Center, Tampere, Finland; Department of Clinical Chemistry, Fimlab Laboratories, Tampere, Finland

[30] Centre for Population Health Research, University of Turku and Turku University Hospital; Research Centre of Applied and Preventive Cardiovascular Medicine, University of Turku; Department of Clinical Physiology and Nuclear Medicine, Turku University Hospital, Turku, Finland

[31] Research Unit of Internal Medicine, Medical Research Center Oulu, Oulu University Hospital, University of Oulu, Oulu, Finland

[32] Faculty of Medicine and Health Technology, Tampere University, and Finnish Cardiovascular Research Center, Tampere, Finland; Department of Clinical Physiology, Tampere University Hospital, Tampere, Finland

***Corresponding author:**

Laura Kananen

laura.kananen@tuni.fi/laura.kananen@ki.se

Online resource 1

Table S1. Description of the variables used in analysis in the MARK-AGE, YFS, and Health 2000

Variable	The Young Finns study, in 2001	Subsample of the Health 2000 Survey, in 2001-2003	MARK-AGE, in 2008-2012
**Smoking**	Daily smoking (0=no, 1=yes)	Have been smoking for at least 100 days and smokes currently (cigarettes, pipe or cigars)? (0=no, 1=yes)	Current smoking (0=no, 1=yes)
**Diet**	Frequency of eating fresh vegetables or roots (0=less than once a month, 1=1-2 times in a month, 2=once a week, 3=couple of times a weel, 4=almost daily, 5=once a day or more)	Frequency of eating fresh vegetables (excluding potatoes) (0=Never, 1=1-2 days/week, 2=3-5 days/week, 3=6-7 days/week)1	Frequency of consuming vegTable Ss (0=Never, 1=1-3 times/month, 2=1-3 times/week, 3=4-6 times/week, 4=Every day, 5=Several times daily)
**Physical activity**	A metabolic equivalent (MET) index: One MET unit = the energy consumption of one kilocalorie per one kilogram of body weight per one hour (Raitakari, Taimela et al. 1996)	Time of physical activity used in commuting and/or the frequency of performing vigorous sports for at least 30 minutes (0=Low level: leisure time vigorous sports not more than once/week and daily less than 30 min walking or biking in commuting, 1=Modest level: leisure time vigorous sports 3 or less times/week and daily less than 30 min walking or biking in commuting, 2=Good level: leisure time vigorous sports more than 3 times/week or daily more than 30 min walking or biking in commuting, 3=High level: leisure time vigorous sports more than 3 times/week and daily more than 30 min walking or biking in commuting)	-
**Physical functioning**	-	Health limits to run 0.5 km (0=no, 1=a little, 2=a lot, 3=completely)	Health limits vigorous activities, such as running, lifting heavy objects and participating in strenuous sports (0=no, 0.5=a little, 1=a lot)
**Number of diseases** (Sum of ten binary items)			
	-	Asthma or chronic obstructive pulmonary disease (0=no, 1=yes)2	Chronic respiratory diseases: chronic obstructive pulmonary disease or asthma (0=no, 1=yes)3
	-	Arthritis, including ostearthritis or rheumatism (0=no, 1=yes)2	Arthritis, including ostearthritis or rheumatism (0=no, 1=yes)3
	-	Osteoporosis (0=no, 1=yes)2	Osteoporosis, brittle bone (0=no, 1=yes)3
	-	Heart failure (0=no, 1=yes)2	Heart failure (0=no, 1=yes)3
	-	Angina pectoris (0=no, 1=yes 2	Angina pectoris (0=no, 1=yes)3
	-	Hypertension (0=no, 1=yes)2	High blood pressure, hypertension treated with prescribed drugs (0=no, 1=yes)3
	-	Diabetes (0=no, 1=yes)2	Diabetes (0=no, 1=yes)3
	-	Cancer/tumor, malignant (0=no, 1=yes)2	Malignant Tumour (0=no, 1=yes)4
	-	Infarction (0=no, 1=yes)2	Myocardial infarction (0=no, 1=yes)4
	-	Stroke, cerebral thrombosis/haemorrhage (0=no, 1=yes)2	Stroke, cerebral thrombosis/haemorrhage (0=no, 1=yes)4

1) within last week, 2) is diagnosed by a medical doctor, 3) health problem/disease in this moment, 4) disease in the past

Table S2. Description of the items used in calculation of frailty index in the MARK-AGE

Item
Able to bathe and dress (0=yes, 1=with limitations, little or lot)
Able to bend, kneel or stoop (0=yes, 1=with limitations, little or lot)
Able to climb several flights of stairs (0=yes, 1=with limitations, little or lot)
Able to lift groceries (0=yes, 1=with limitations, little or lot)
Able to perform chair rise test (0=yes, 1=not at all or stop after 1-4 times)
Angina pectoris (0=no, 1=yes)
Arthritis (0=no, 1=yes)
Backpain (0=no, 1=yes)
Chronic respiratory disease (chronic obstructive pulmonary disease or asthma; 0=no, 1=yes)
Diabetes (0=no, 1=yes)
Falls (1=yes, 0=no)[2]
Feeling down¬hearted and low (0=never or seldom, 0.5= some amount of time or good bit of time, 1=all the time or most of the time)[3]
Feeling full of life (1=never or seldom, 0.5= some amount of time or good bit of time, 0=all the time or most of the time)[3]
Feeling happy (1=never or seldom, 0.5= some amount of time or good bit of time, 0=all the time or most of the time)[3]
Feeling nervous (0=never or seldom, 0.5= some amount of time or good bit of time, 1=all the time or most of the time)[3]
Feeling so down that nothing that nothing cheers up (0=never or seldom, 0.5=some amount of time or good bit of time, 1=all the time or most of the time)[3]
Feeling tired (0=never or seldom, 0.5= some amount of time or good bit of time, 1=all the time or most of the time)[3]
Feeling worn out (0=never or seldom, 0.5= some amount of time or good bit of time, 1=all the time or most of the time)[3]
Health limits moderate activities, such as moving a table, pushing a vacuum cleaner, bowling or playing golf (0=no, 0.5=a little, 1=a lot)
Health limits walking more than a mile (0=no, 0.5=a little, 1=a lot)
Health limits vigorous activities, such as running, lifting heavy objects and participating in strenuous sports (0=no, 0.5=a little, 1=a lot)
Hearing problem (0=no, 1=yes)
Heart failure (0=no, 1=yes)
High cholesterol (0=no, 1=yes)
Hypertension (treated with prescribed drugs) (0=no, 1=yes)
Hyperthyroidism (0=no, 1=yes)
Hypothyroidism (0=no, 1=yes)
Irregular heartbeat (0=no, 1=yes)
Leg pain during walking (0=no, 1=yes)
Neurological disease (e.g. Parkinson's disease; 0=no, 1=yes)
Osteoporosis (0=no, 1=yes)
Pain at different locations (0=no, 1=yes)
Malignant Tumour (0=no, 1=yes)[1]
Myocardial infarction (AMI) (0=no, 1=yes)[1]
Stroke, cerebral thrombosis/haemorrhage (0=no, 1=yes)[1]
Physical health or emotional problems interfered normal social activities (0=not at all or slightly, 0.5=moderately, 1=quite a bit or extremely[3]
Self-rated health status (0=excellent, 0.33=very good, 0.67=good, 1=fair or poor)
Statement: more easily ill than others (0=definitely or mostly false, 0.5=not true, 1=definitely or mostly true)
Venous insufficiency in legs/leg ulcers (0=no, 1=yes)

1) disease in the past, 2) within last year, 3) within last two weeks

Table S6. Summary of the laboratory methods used to assess the biomarker levels in the MARK-AGE

**Methods**	**Sum of biomarkers**
NMR, nuclear magnetic resonance technology	31
Beckman access	28
DSA-FACE, DNA sequencer-assisted flurophore-assisted carbohydrate electrophoresis	19
HPLC, high-performance liquid chromatography	16
ELISA, enzyme-linked immunosorbent assay	9
FACS, fluorescence-activated cell sorting	8
HPLC-ICP-MS, A high-performance liquid chromatography in combination with inductively coupled plasma mass spectrometry	6
ICP-MS, inductively coupled plasma mass spectrometry	5
AutoDELFIA time-ressolved fluorescence immunoassay	4
Formula	3
Other	13
**Total**	**142**

Table S7. Summary of the laboratory methods used to assess the biomarker levels and biomarker domains in the analysis in the sample of YFS (n=1701)

**Method**	**Domain**	**Sum of biomarkers**
NMR, nuclear magnetic resonance technology	Lipoprotein subclasses	71
	Lipid extract metabolites	24
	Low-molecular-weight metabolites	21
	Lipids, Combined measures	11
	Lipids, Values estimated with the Extended Friedewald method	8
	Glucose and lipid metabolism	1
	Transporter protein	1
Other	Lipids	6
	Glucose metabolism	1
	Inflammation	1
	Tissue-damage	1
	One-carbon metabolism	1
	**Total**	**147**

Table S8. Summary of the laboratory methods used to assess the biomarker levels and the biomarker domains in the analysis in the Health 2000 (n=1196)

Method	Domain	Sum of biomarkers
NMR, nuclear magnetic resonance technology	Lipids	208
	Low-molecular-weight metabolites	19
Other	Lipid metabolism	5
	Regulator of energy metabolism	4
	Inflammation	3
	Tissue-damage	1
	Glucose metabolism	1
	**Total**	**241**

Table S9. Biomarker domains analysed in the sample of MARK-AGE (n=1479)

Domain	Sum of biomarkers
Lipid metabolism	28
Protein modification	21
Immune system	19
Nutrition	16
Iron metabolism	7
Oxidative stress	5
Lipid oxidation product; oxidative stress	4
Oxygen transfer	4
Aminoacid metabolism	3
Byproduct of muscle metabolism	3
Copper metabolism	3
Glucose metabolism	3
Selenium metabolism	3
One carbon cycle	3
Metabolite	3
Protein metabolism	2
Pyrimidine metabolism	2
Amino acid metabolism	1
Copper and iron metabolism	1
Copper and zinc metabolism	1
Endocrine functions	1
Glucose and lipid metabolism	1
Neurotransmitter	1
Metabolism of nitrogen-containing compounds	1
Antiprotease	1
Purine metabolism	1
Tissue damage	1
Tissue revascularization, wound healing, and tissue repair	1
Zinc metabolism	1
Transporter protein	1
**Total**	**142**

Table S10. The relationship of cf-DNA levels to age, sex, health behaviour and ageing phenotypes

Age, sex, smoking, vegetable consumption, physical activity, physical functioning, number of diseases, and frailty were analysed one by one, using simple linear regression, in the MARK-AGE, YFS, and Health 2000, and also stratified by sex. Bonferroni-adjusted p-values<0.05 are bolded.

			Variable							
**Data set**			**Age**	**Sex**	**Smoking**	**Vegetable consumption**	**Physical activity**	**Physical functioning**	**Number of diseases**	**Frailty index**
**MARK-AGE**	**All**	**β**	0.000354	-0.0749	0.0363	-0.0184	-	0.0133	0.00782	0.0635
		**p**	0.180	**1.02x10^{-39}**	**3.59x10^{-06}**	**1.59x10^{-09}**	-	0.0931	0.0126	0.0171
	**Women**	**β**	0.000477	-	0.030	-0.00906	-	0.0173	0.0144	0.131
		**p**	0.142	-	0.00244	0.0204	-	0.0725	**0.000151**	**0.0000199**
	**Men**	**β**	-0.0000954	-	0.0327	-0.0150	-	0.0276	0.00515	0.114
		**p**	0.812	-	0.00420	0.00101	-	0.0250	0.288	0.0119
**YFS**	**All**	**β**	0.002518	-0.0587	0.00929	-0.00574	0.0000278	-	-	-
		**p**	0.00540	**8.10x10^{-11}**	0.388	0.100	0.907	-	-	-
	**Women**	**β**	0.00500	-	-0.02280	-0.00249	-0.000013	-	-	-
		**p**	**0.0000987**	-	0.174	0.638	0.971	-	-	-
	**Men**	**β**	-0.000630	-	0.020	-0.001045	0.000015	-	-	-
		**p**	0.603	-	0.131	0.817	0.959	-	-	-
**Health 2000**	**All**	**β**	0.00058	-0.0822	0.0593	-0.0134	-0.0104	0.00258	0.00781	-
		**p**	0.174	**6.40x10^{-35}**	**9.75x10^{-12}**	**0.000530**	0.00482	0.338	0.0250	-
	**Women**	**β**	0.00178	-	0.0306	-0.0108	-0.00806	0.00693	0.0110	-
		**p**	**0.000189**	-	0.00507	0.0296	0.0560	0.0230	0.00601	-
	**Men**	**β**	-0.00117	-	0.0676	-0.00478	-0.00831	0.00854	0.00483	-
		**p**	0.0863	-	**6.79x10^{-08}**	0.385	0.155	0.0533	0.377	-

Table S11. The relationship of cf-DNA levels to sex, age, and health behaviors in the multivariate analysis

In model 1, age, sex, smoking, and vegetable consumption, and in model 2 also physical activity were analysed using multivariate linear regression. The analysis was performed in the full samples of the MARK-AGE, YFS, and Health 2000, and also stratified by sex. Bonferroni-adjusted p-values<0.05 are bolded.

					Variable					
**Model**	**Sample**		**n**		**Sex**	**Age**	**Smoking**	**Vegetable consumption**	**Physical activity**	**R^2**
1	**MARK-AGE**	**All**	2261	**β**	-0.0699	0.000468	0.0300	-0.0111	-	0.0876
				**p**	**3.05×10^{-34}**	0.0682	**8.65×10^{-05}**	**0.0002**	-	
		**Women**	1225	**β**		0.000653	0.0302	-0.0081	-	0.0138
				**p**		0.0456	0.00285	0.0400	-	
		**Men**	1036	**β**		0.000274	0.0297	-0.0141	-	0.0168
				**p**		0.502	0.0104	0.0024	-	
	**YFS**	**All**	1928	**β**	-0.0574	0.00258	-0.0011	-0.003	-	0.026
				**p**	**4.69×10^{-10}**	0.00418	0.917	0.4077	-	
		**Women**	1072	**β**		0.00516	-0.0246	-0.0064	-	0.0169
				**p**		**6.79×10^{-05}**	0.1449	0.23	-	
		**Men**	856	**β**		-0.000597	0.0205	0.0009	-	0.00298
				**p**		0.624	0.1349	0.8493	-	
	**Health 2000**	**All**	1196	**β**	-0.0772	0.000929	0.0511	-0.0047	-	0.151
				**p**	**1.74×10^{-31}**	0.0194	**1.03×10^{-09}**	0.1924	-	
		**Women**	683	**β**		0.002	0.0372	-0.0084	-	0.0421
				**p**		**3.43×10^{-05}**	**0.000675**	0.087	-	
		**Men**	513	**β**		-0.00071	0.066	0.0001	-	0.0575
				**p**		0.29	**2.67×10^{-07}**	0.9824	-	
2	**YFS**	**All**	1928	**β**	-0.0571	0.00264	-0.00054	-0.0033	0.000117	0.0261
				**p**	**6.17×10^{-10}**	0.00371	0.961	0.371	0.632	
		**Women**	1072	**β**		0.00523	-0.0242	-0.0068	0.00017	0.0171
				**p**		**6.16×10^{-05}**	0.1529	0.2106	0.651	
		**Men**	856	**β**		-0.000546	0.0212	0.0007	0.0000844	0.00307
				**p**		0.657	0.1287	0.891	0.782	
	**Health 2000**	**All**	1196	**β**	-0.077	0.000946	0.0497	-0.004	-0.0053	0.153
				**p**	**2.49×10^{-31}**	0.0173	**3.36×10^{-09}**	0.2742	0.127	
		**Women**	683	**β**		0.001972	0.0362	-0.0075	-0.00559	0.0446
				**p**		**4.23×10^{-05}**	**0.00097**	0.1271	0.182	
		**Men**	513	**β**		-0.000685	0.0654	0.0003	-0.00171	0.0577
				**p**		0.311	**4.75×10^{-07}**	0.954	0.771	

Table S12. The relationship of cf-DNA levels to sex, age, health behaviors and ageing phenotypes in the multivariate analysis
In model 3, age, sex, smoking, and vegetable consumption, physical function and number of diseases and in model 4, frailty instead of diseases and physical functioning were analysed using multivariate linear regression. The analysis was performed in the full samples of the MARK-AGE and Health 2000, and also stratified by sex. Bonferroni-adjusted p-values<0.05 are bolded.

					Variable							
**Model**	**Sample**		**n**		**Sex**	**Age**	**Smoking**	**Vegetable consumption**	**Physical functioning**	**Number of diseases**	**Frailty**	R^2
**3**	**MARKAGE**	**All**	2261	**β**	-0.0722	-0.00000017	0.0283	-0.0108	0.0122	0.00938	-	0.0926
				**p**	**$5.37x10^{-36}$**	0.9995	**0.000224**	**0.000336**	0.1387	0.00619	-	
		**Women**	1225	**β**	-	0.0000936	0.0283	-0.00763	0.00314	0.0136	-	0.0237
				**p**	-	0.801	0.00530	0.0516	0.761	0.00178	-	
		**Men**	1036	**β**	-	-0.0000976	0.0275	-0.0139	0.0238	0.00423	-	0.0214
				**p**	-	0.829	0.0181	0.00269	0.0711	0.439	-	
	**Health 2000**	**All**	1196	**β**	-0.0792	0.000394	0.0501	-0.00353	0.00391	0.00549	-	0.155
				**p**	**$7.15x10^{-32}$**	0.388	**$2.19x10^{-09}$**	0.338	0.187	0.141	-	
		**Women**	683	**β**	-	0.00175	0.0371	-0.00796	0.0000442	0.00461	-	0.0436
				**p**	-	0.00190	**0.000713**	0.109	0.990	0.312	-	
		**Men**	513	**β**	-	-0.00149	0.0631	0.00199	0.00885	0.00486	-	0.0675
				**p**	-	0.0480	**$9.25x10^{-07}$**	0.718	0.0883	0.437	-	
**4**	**MARKAGE**	**All**	2261	**β**	-0.0746	0.000119	0.0265	-0.0104	-	-	0.112	0.0944
				**p**	**$1.99x10^{-37}$**	0.658	**0.000542**	**0.000569**	-	-	**$4.18x10^{-05}$**	
		**Women**	1225	**β**	-	0.000263	0.0267	-0.00682	-	-	0.114	0.0237
				**p**	-	0.442	0.00845	0.0822	-	-	**0.000440**	
		**Men**	1036	**β**	-	-0.0000280	0.0263	-0.0139	-	-	0.109	0.0237
				**p**	-	0.948	0.0240	0.00270	-	-	0.0206	

Table S13. The relationship of cf-DNA levels to sex, age, health behaviors and frailty in three age groups in the MARK-AGE assessed using multivariate linear regression
Bonferroni-adjusted p-values<0.05 are bolded.

				Variables in the model					
	**Age group, years**	**n**		**Sex**	**Age**	**Smoking**	**Vegetables**	**Frailty index**	R^2
**MARK-AGE**	**<47**	440	**β**	-0.0861	-0.000224	-0.00342	-0.00977	0.0468	0.105
			**p**	**$9.29x10^{-10}$**	0.898	0.826	0.133	0.616	
	**47-65**	1096	**β**	-0.0685	0.00117	0.0524	-0.0102	0.0952	0.0987
			**p**	**$1.77x10^{-15}$**	0.155	**$2.6\ x10^{-06}$**	0.0250	0.0207	
	**>65**	725	**β**	-0.0748	0.00222	0.00508	-0.00922	0.133	0.105
			**p**	**$5.25x10^{-15}$**	0.136	0.738	0.0672	**0.000595**	

Table S14. Biomarker profiles for the cf-DNA level
Regression coefficients and their unadjusted p-values from the multivariate linear regression are shown for **A)** MARK-AGE, **B)** YFS, and **C)** Health 2000, and stratified by sex.

Abbreviations: CRP = C-reactive protein, HDL = high density lipoprotein, LDL = low density lipoprotein, MDA = malondialdehyde, VLDL = very low density lipoprotein
p-value=0: p-value<$1x10^{-16}$

**A.**	**All**		**Women**		**Men**	
	**β**	**p**	**β**	**p**	**β**	**p**
Sex	-0.0267	0.000808	-	-	-	-
Age	0.000123	0.676	-0.000420	0.269	0.000541	0.252
Plasma, fibrinogen	0.0100	0.00208	0.0112	0.00563	0.00624	0.234
Plasma, uric acid	0.0106	0.00292	0.0141	0.00325	0.00675	0.212
Serum, ratio of peak 7 N-glycan to total proteins	0.00539	0.0798	0.00379	0.320	0.00662	0.185
Serum, ratio of peak 8 N-glycan to total proteins	0.0113	0.00261	0.0113	0.0126	0.00974	0.123
Serum, ratio of peak 9 N-glycan to total proteins	0.0113	0.00203	0.0105	0.0237	0.0130	0.0248
Serum, proteins (total)	0.0128	0.000816	0.0142	0.00270	0.0119	0.0568
Plasma, protein-bound 3-nitrotyrosine	0.00760	0.0130	0.00945	0.00343	0.000435	0.950
Serum, MDA/LDL protein	-0.00904	0.00291	-0.00858	0.0148	-0.00778	0.149
Serum, apolipoprotein J/clusterin	0.0138	0.00000785	0.00868	0.0123	0.0212	0.000
Plasma, homocysteine	0.0116	0.000334	0.0138	0.00115	0.0103	0.0382
Plasma, ascorbic acid	-0.00953	0.00287	-0.00843	0.0153	-0.0120	0.0624
Plasma, carotenoid beta-cryptoxanthine	-0.00954	0.00279	-0.00622	0.0838	-0.0156	0.00851
Plasma, carotenoid lutein	-0.0108	0.000610	-0.00626	0.0862	-0.0149	0.00703
Urine, relative trigonelline	-0.00685	0.0269	-0.00388	0.252	-0.0127	0.0412
Plasma, selenium (eluting with retention time of Albumin or Selenoprotein P)	-0.0166	0.0000000742	-0.0137	0.00052	-0.0179	0.000259
Serum, Fe	0.0152	0.000000824	0.00775	0.03651	0.0244	0.00000233
Plasma, CRP	0.0174	0.000000333	0.0170	0.000153	0.0191	0.000278
Serum, immunoglobulin G	-0.0113	0.00648	-0.00714	0.171	-0.0148	0.0258
Serum, glucose	-0.0123	0.000100	-0.000350	0.928	-0.0270	0.000000259
Plasma, Ratio of copper to zinc	-0.0296	0	-0.0319	0	-0.0248	0.00142
**R^2**		**0.258**		**0.199**		**0.213**

**B.**	**All**		**Women**		**Men**	
	**β**	**p**	**β**	**p**		**p**
Sex	-0.0470	0.0000183	-	-	-	-
Age	0.00360	0.0000554	0.00404	0.00122	0.00151	0.234
Albumin	0.0235	0.00000696	0.0225	0.00125	0.0203	0.0106
3-hydroxybutyrate	0.0473	0.00000000393	0.0107	0.000334	0.0503	0.0000545
Acetoacetate	-0.0243	0.00144	-0.0141	0.222	-0.0333	0.000984
Cholesterol esters in large HDL	0.0237	0.000535	0.0137	0.142	0.0373	0.000306
Total lipids in very large VLDL	-0.0059	0.762	0.0141	0.650	-0.0116	0.630
Chylomicrons and extremely large VLDL particles	0.128	0.00000000000000784	0.163	0.00000000000526	0.0903	0.0000561
Phospholipids in chylomicrons and extremely large VLDL	-0.106	0.0000000875	-0.153	0.00000307	-0.0843	0.000574
Ratio of bisallylic groups to double bonds	0.0315	0.00000000533	0.0487	0.000000000432	0.00928	0.200
Sphingomyelins	-0.0109	0.0894	-0.0169	0.0487	-0.00137	0.885
Phosphoglycerides, total	-0.0525	0.00000000428	-0.0567	0.00000124	-0.0243	0.0966
Citrate	0.0186	0.000206	0.0277	0.0000426	0.00497	0.496
Isoleucine	-0.0510	0.0000000958	-0.0575	0.0000628	-0.0433	0.000407
Leucine	0.0756	0	0.0742	0.000000000170	0.0778	0.0000000000304
Tyrosine	0.0135	0.0147	0.0191	0.0105	0.0054	0.502
**R^2**		**0.190**		**0.222**		**0.153**

**C.**	**All**		**Women**		**Men**	
	**β**	**p**	**β**	**p**		**p**
Sex	-0.0804	0	-	-	-	-
Age	0.0000964	0.804	0.000443	0.361	-0.000670	0.296
Glycoprotein acetylation	0.0560	0	0.0562	0.000000000000293	0.0470	0.0000000430
Creatinine	0.0101	0.00887	0.0215	0.000111	0.00460	0.397
Cholesterol, total	0.0379	0	0.0422	0.0000000000000530	0.0257	0.000326
3-hydroxybuturate	0.0143	0.00000268	0.00956	0.0118	0.0194	0.0000777
Ratio of 18:2 linoleic acid to total fatty acids	-0.0226	0.00000000123	-0.0109	0.0238	-0.0303	0.000000217
Saturated fatty acids	-0.0598	0	-0.0726	0	-0.0402	0.000156
Estimated degree of unsaturation	-0.0139	0.0000942	-0.00718	0.104	-0.0177	0.00221
Phenylalanine	-0.00847	0.0578	-0.0100	0.0911	-0.00685	0.310
Histidine	-0.00733	0.0568	-0.0113	0.0195	-0.00379	0.541
Isoleucine	-0.0495	0.00000000116	-0.0396	0.000280	-0.0480	0.0000813
Leucine	0.0268	0.00127	0.0280	0.0153	0.0218	0.0693
Glycine	0.00954	0.00470	0.00703	0.0497	0.00694	0.404
Tyrosine	0.0107	0.00689	0.0241	0.00000636	-0.00150	0.800
**R^2**		**0.233**		**0.176**		**0.163**

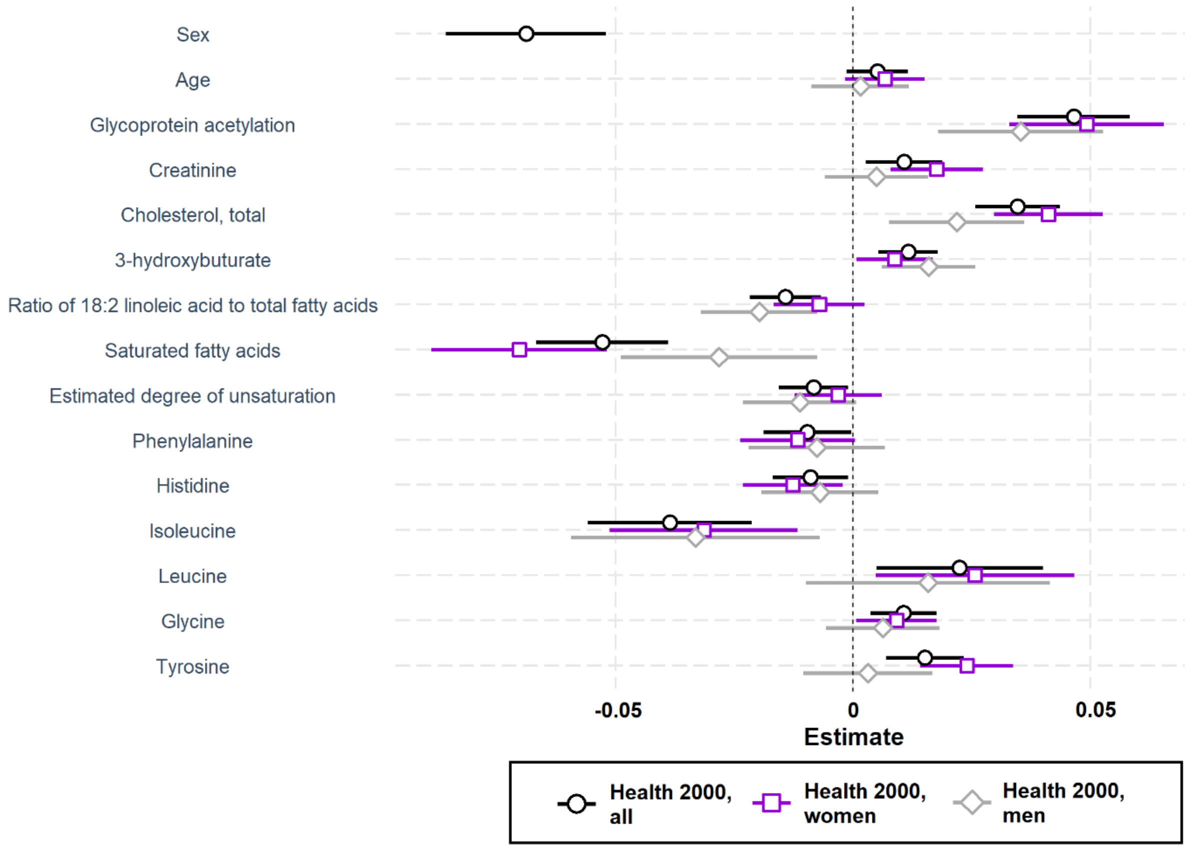

Fig S4. Biomarker profile related to cf-DNA levels in non-smokers
Metabolic profile identified in the full analytical sample in the Health 2000 (Fig4c, Table S14c) was additionally tested in non-smokers of the same cohort, also stratified by sex. The results are presented as forest plot in which regression coefficients and their unadjusted confidence intervals (95% CIs, as whiskers) are shown.

References

RAITAKARI, O.T., TAIMELA, S., PORKKA, K.V.K., LEINO, M., TELAMA, R., DAHL, M. and VIIKARI, J.S.A., 1996. Patterns of intense physical activity among 15- to 30-year-old Finns. *Scandinavian Journal of Medicine & Science in Sports*, **6**(1), pp. 36-39.
